# A Cross-Sectional Study on the Epidemiology of Newly Diagnosed Breast Cancer Patients Attending Tertiary Care Hospitals in a Tribal Preponderant State of India: Regression Analysis

**DOI:** 10.7759/cureus.40489

**Published:** 2023-06-15

**Authors:** Zenith H Kerketta, Anit Kujur, Neelanjali Kumari, Vidya Sagar, FNU Pushpa

**Affiliations:** 1 Surgery, Rajendra Institute of Medical Sciences, Ranchi, IND; 2 Community Medicine, Rajendra Institute of Medical Sciences, Ranchi, IND; 3 Preventive Medicine, Mahatma Gandhi Memorial Medical College, Jamshedpur, Jamshedpur, IND; 4 Preventive and Social Medicine, Rajendra Institute of Medical Sciences, Ranchi, IND; 5 Preventive Medicine, Rajendra Institute of Medical Sciences, Ranchi, IND

**Keywords:** newly diagnosed, regression anallysis, tribal, epidemiology, breast cancer

## Abstract

Introduction: Breast cancer (BC) is globally prevalent and the leading cause of death due to cancer in females. Due to changes in risk factor profiles, improved cancer registration, and cancer detection, its incidence and death rates have risen over the past three decades. Both modifiable and immutable risk factors for BC make up a sizable portion of the total risk factors.

Methodology: This was a hospital-based cross-sectional study carried out in the Department of Surgery, Rajendra Institute of Medical Sciences (RIMS), Ranchi. Consecutive sampling was done with a complete enumeration of all newly diagnosed cases of breast cancer >15 years old. Those who consented to participate and those who were extremely ill, deaf, or dumb were excluded from the study.

Results: A total of 88 patients were included. Maximum patients diagnosed with breast cancer belonged to the age group of 40-50 years (37.5%), Hindu by religion (76.1%), non-tribal (80.68%), illiterate (89.8%), married (98.9%), housewives (92%), and of class IV socio-economic status (SES) (65.9%).

Conclusion: Regular training of Sahiya (the local name of Accredited Social Health Activist (ASHA) in Jharkhand), empowerment of screening clinics for cancer, and upgraded diagnostic facilities for timely referral should be stressed upon.

## Introduction

Breast cancer is the most prevalent cancer and the foremost cause of cancer death among women across the globe [1-3]. Asia accounts for 50% of all breast cancer deaths worldwide and 43% of new cases diagnosed each year [1]. With an estimated 2.3 million new cases in 2020 or 11.7% of all cancer cases, breast cancer in females has completely surpassed lung cancer as the leading cause of cancer incidence worldwide [4]. Breast cancer is the most common cancer in India, with a prevalence rate of 25.8 cases per 100,000 people, and in 2020, it will account for 13.6% of all new cancer cases and roughly 13.3 fatalities per 100,000 people in 2020 [4]. Approximately 13.3 deaths per 100,000 people in 2020. The survival rate for breast cancer has also dropped 2.7 times in cases of detection at stage IV as against stage I, and almost 90,408 females died on account of this in 2020 in India [5]. Breast cancer is still the most common cause of cancer death among women in developing regions [6]. The prevailing belief about the occurrence of cancer is conventionally smoking or drinking. But the fact is that it is multifarious, and if detected early, it can be managed in a much-favorable way with a good prognosis and with even much less mortality. Also, there is much scientific evidence that advocates that 30% of cancers are curable if detected prior and prompt treatment is provided, 40% are preventable, and the rest of the 30% of advanced cancers are looked after with palliative care because of the need for efficacious pain relief measures [7]. The National Cancer Registry Program was started in 1981 in India to generate data on the magnitude and its patterns of cancer through population-based registries [8,9], but still Jharkhand remains out of the loop as it lacks a robust registry system to list the number of patients and classify the forms of the disease [10]. The oncologists from various cities in Jharkhand imply an enormous upsurge in the cases documented per year even without any centralized data [10].

For the formulation and planning of befitting cancer strategies based on scientific and empirical statistics, absolute knowledge of epidemiology is a sine qua non for policymakers and authorities. There have been previous attempts to describe the national-level distribution of cancer burden and epidemiology in different parts of India as well as implications for cancer control [11-25], but a systematic and comprehensive understanding of the enormity and time trends of multiplex cancers in each state of India is not readily accessible. Practical availability of screening programs and treatment for breast cancer and upgraded health awareness would be advantageous, beneficial, and a great aid for initiating various cancer control programs for the inhabitants of Jharkhand since health care delivery is a subject of the respective state in India. Thereupon, an attempt was made to do this novel study to know the magnitude of breast cancer patients with respect to time, place, and person in Jharkhand so that we could acquire the exact picture of breast cancer in Ranchi. 

## Materials and methods

This was a hospital-based cross-sectional analytical study done in the Department of Surgery at Rajendra Institute of Medical Sciences, Ranchi, Jharkhand. All newly diagnosed confirmed cases of breast cancer in females admitted to the Surgery Department at RIMS, Ranchi, were taken as the study population using the consecutive sampling technique, which was a convenient method. Newly diagnosed confirmed cases are females who found lumps, or if an area of the breast looks abnormal on breast self-examination (BSE), then breast mammography was done among them. For confirmation and staging, a biopsy was carried out among them. All newly diagnosed females with breast cancer >15 years of age and those who were willing to participate and gave consent for the study were included in the study. Critically ill patients and deaf and dumb patients were excluded. The duration of the study was 12 months (January 2019-December 2019). Ethical approval for the study was obtained from the Institutional Ethics Committee of Rajendra Institute of Medical Sciences (RIMS), Ranchi, with No. 08/IEC/RIMS, February 16, 2019. Interviews with study subjects were conducted in hospitals after proper written informed consent in Hindi. The subjects were explained about the purpose of the study, which was to know the magnitude of disease among them and also how early detection will help in the cure of disease with timely intervention. A semi-structured questionnaire was used for data collection, which was pre-tested before the data collection to validate it. A proper template was generated for data entry in an MS Excel sheet (Microsoft version 2013, Microsoft Corporation, Redmond, Washington, USA). The data were analyzed using the software Statistical Package for Social Science (SPSS, version 25.0; IBM Corp., Armonk, NY, USA). Binary logistic regression was used to determine the predictors of breast cancer and the significant association. For statistical analysis, a p-value <0.05 was considered significant.

## Results

The present study was done among 88 newly diagnosed female breast cancer patients. The majority of the patients were in the age group of 40-50 years, which constitutes about 37.5% of the total study participants. They were Hindu by religion (76.1%), non-tribal (80.68%), illiterate (89.8%), married (98.9%), housewives (92%), and of class IV socio-economic status according to the modified B.G. Prasad's classification 2020 (65.9%) (Table 1).

**Table 1:** Sociodemographic characteristics of breast cancer patients (n=88).

Characteristics		Frequency	Percentage
Age in years	<30	4	4.5
	30-40	10	11.4
	40-50	33	37.5
	50-60	23	26.1
	>60	18	20.5
Religion	Hindu	67	76.1
	Muslim	09	10.2
	Christian	05	5.7
	Sarna	07	8
Ethnicity	Tribal	17	19.3
	Non-tribal	71	80.68
Education	Illiterate	79	89.8
	Primary	03	3.4
	Middle	03	3.4
	Secondary	03	3.4
Occupation	Student	01	1.1
	Housewife	81	92
	Daily wage worker	04	4.5
	Government job	01	1.1
	Others	01	1.1
Marital status	Married	87	98.9
	Unmarried	01	1.1
Socio-economic status (according to B.G. Prasad's classification)	Class I	01	1.14
	Class II	05	5.7
	Class III	14	15.9
	Class IV	58	65.9
	Class V	10	11.4

Binary logistic regression was applied to determine the significant association between the predictors of breast cancer. On applying logistic regression, age at diagnosis was 9.46 times associated with breast cancer, but the factor was not found to be significant with a P-value of 0.18. For age at menarche, the early age of menarche was found to be 2.89 times associated with breast cancer, but the factor was not found to be statistically significant with a P-value of 0.20. Age at first childbirth, religion, education, occupation, and present status of menstruation were also not found to be significantly associated with breast cancer. Ethnicity showed that breast cancer was 2.37 times more significantly associated among the tribals as compared to the non-tribals with a P-value of 0.02 and a 95% confidence interval of (1.42-81.71) (Table 2).

**Table 2:** Binary logistic regression for the predictors of breast cancer patients. AOR: adjusted odds ratio.

Variables	B	SE	AOR	95% CI for odds ratio, lower/upper		P-value
Age at diagnosis						
<40	2.24	1.59	9.46	0.4	74.64	0.18
>40	Reference					
Age at menarche						
<14	1.062	0.830	2.89	0.568	14.715	0.20
>14	Reference					
Age at first childbirth						
<16	1.037	0.702	2.18	0.713	11.15	0.14
>16	Reference					
Religion						
Hindu	0.115	0.982	0.892	0.130	6.108	0.90
Non-Hindu	Reference					
Ethnicity						
Tribal	2.37	1.03	10.77	1.42	81.71	0.02
Non-tribal	Reference					
Education						
Illiterate	0.416	1.201	1.516	0.144	15.95	0.72
Literate	Reference					
Occupation						
Working	−0.191	1.23	0.826	0.074	9.22	0.87
Housewife	Reference					
Present status of menstruation						
Menstruating	−0.255	0.704	0.775	0.195	3.079	0.71
Menopause	Reference					

## Discussion

In this present study, the majority of the females were illiterate housewives between the age group of 40-50 years, belonging to the Hindu religion and of non-tribal ethnicity. Most patients were married and had more than three children. Most of the patients were postmenopausal and most of them had their menarche at the age of >13 years (Table 1). The majority of the patients in this study were in the age group of 40-50 years, which was similar to studies done by Singh et al., which also showed the same results. Various other studies [26-28] done on Indian women having breast cancer found that they were a decade younger in comparison to western women, suggesting that breast cancer occurs at a younger pre-menopausal age in India, which is quite similar to the result of this study.

The majority of the patients (97.7%) in this present study had their menarche at age >14 years, which was similar to the study done by Sandhu et al., where younger age during menarche was not associated with an increased risk of breast cancer [29-31]. Among the breast cancer patients admitted to RIMS, the majority of the patients had menarche at 14 years of age and >14 years of age; both were diagnosed with locally advanced breast cancer (LABC) and advanced stage. A study in Italy showed no relationship between breast cancer and the duration of menstruation cycles [31]. Whereas the result of a large population study among 11,889 women in China showed that a younger age of menarche is associated with an increased risk of breast cancer among them (95% CI, 1.1-3.4) [32]. The findings of a case-control study indicated that younger age during menarche increases the risk of breast cancer by two times (OR, 2.83; 95% CI, 1.02-7.86) [32].

In this study, 73.9% of the participants were postmenopausal females. Among the breast cancer patients admitted to RIMS, the majority were either menstruating or post-menopausal; both were diagnosed in LABC and advanced stages. Studies done by Fooretti et al. also showed that the age of menopause over 50 years was associated with an increased risk of breast cancer [33-36]. The results of a case-control study done by Thakur et al. also confirmed the association between older age in menopause and the incidence of breast cancer (OR, 2.43; 95% CI, 1.2-4.9) [35].

The age at first childbirth in the present study of the majority of the patients was <18 years. Among the breast cancer patients admitted to RIMS, the majority of the patients had their first childbirth at 16 years of age and >16 years of age, both were diagnosed with LABC and advanced stages.

In a case-control study, older age during the first childbirth was the most important risk factor for breast cancer, with a relative risk of more than six times (OR, 6.34; 95% CI, 2.04-27) [33]. Another study indicated that every childbirth reduces the risk of progesterone receptor (PR+) and estrogen receptor (ER+) cancers by up to 10% (risk ratio (RR) per birth, 0.89; 95% CI, 0.84-0.94), and women who were older at their first childbirth had a 27% increased risk of developing breast cancer (RR, 1.27; 95% CI, 1.07-1.50) [37,38]. In a study done by Laamiri et al., it was found that, in addition to full-term pregnancy, early maternal age reduces the risk of developing breast cancer by up to 23% [38]. Another study done by Kim et al. showed a positive correlation between the age of more than 26 years during the first childbirth and lobular disease (OR, 1.35; 95% CI, 1.03-1.78) [36]. Several other studies found that older age at first full-term pregnancy was associated with an increased risk of developing breast cancer [35]. In a study done by Dai et al., the first full-term pregnancy in women aged 20 years or older was associated with 40%-50% increased risk of breast cancer [39-41]. 

On applying logistic regression, no significant difference in terms of age at diagnosis, age at menarche, age at first childbirth, religion, education, occupation, or present status of menstruation was found, and only ethnicity showed that breast cancer was 2.37 times more common among the tribals as compared to non-tribals, and it was found to be significant with a P-value of 0.02 (95% CI, 1.42-81.71) (Table 2). It was contradictory to the findings were seen by Fioretti et al., where educational level and employment status (OR, 0.32; 95% CI, 0.19-0.56) are among the most important socioeconomic variables that affect the incidence rate of breast cancer [33]. Researchers believe that employed women generally have a higher income and are more likely to use health insurance. Furthermore, the economic situation can contribute to a person’s willingness to spend money on medical care, but the socioeconomic status was not found to be significantly associated. Due to differences in lifestyle, diet, and environmental factors, living in urban areas is associated with an increase in the risk of developing breast cancer [42], but this was not assessed in our study due to time constraints and a paucity of resources. Socioeconomic status is associated with the clinical course and survival rate (7% absolute difference in overall survival, P≤0.001; 4% cancer-specific survival, P≤0.001) [43-46].

Limitation

As the sample size was very small and the study was not multicentric (done only in one hospital RIMS, Ranchi) and was a cross-sectional study, the temporal association of determinants of breast cancer could not be established. A hypothesis was generated by this descriptive study, and to test this hypothesis, further studies like case-control studies are required.

Further lifestyle, diet, and environmental factors indicate that living in urban areas is associated with an increase in the risk of developing breast cancer, but this was not assessed in our study due to time constraints and a paucity of resources.

## Conclusions

Our study concluded that tribal females were significantly associated with breast cancer, so education and awareness about breast cancer and BSE should be included in the educational curriculum so that school-going girls are educated at a young age. Regular training in breast self-examination (BSE) should be given to ASHA so that early diagnosis can be made at the community level and timely referrals can be made for proper intervention at the hospitals. Various training sites for IEC and training of female patients for BSE should be in the different wards of hospitals. Non-communicable disease screening clinics, especially for breast cancer, should be opened at the primary level so that diagnostic facilities are also available for timely referral to higher facilities among tribals who live in hard-to-reach areas. 
